# Recent Progress of Imprinted Nanomaterials in Analytical Chemistry

**DOI:** 10.1155/2018/8503853

**Published:** 2018-07-02

**Authors:** Rüstem Keçili, Chaudhery Mustansar Hussain

**Affiliations:** ^1^Anadolu University, Yunus Emre Vocational School of Health Services, Department of Medical Services and Techniques, 26470 Eskişehir, Turkey; ^2^Department of Chemistry and Environmental Science, New Jersey Institute of Technology, Newark, N J 07102, USA

## Abstract

Molecularly imprinted polymers (MIPs) are a type of tailor-made materials that have ability to selectively recognize the target compound/s. MIPs have gained significant research interest in solid-phase extraction, catalysis, and sensor applications due to their unique properties such as low cost, robustness, and high selectivity. In addition, MIPs can be prepared as composite nanomaterials using nanoparticles, multiwalled carbon nanotubes (MWCNTs), nanorods, quantum dots (QDs), graphene, and clays. This review paper aims to demonstrate and highlight the recent progress of the applications of imprinted nanocomposite materials in analytical chemistry.

## 1. Introduction

**Figure 1:**
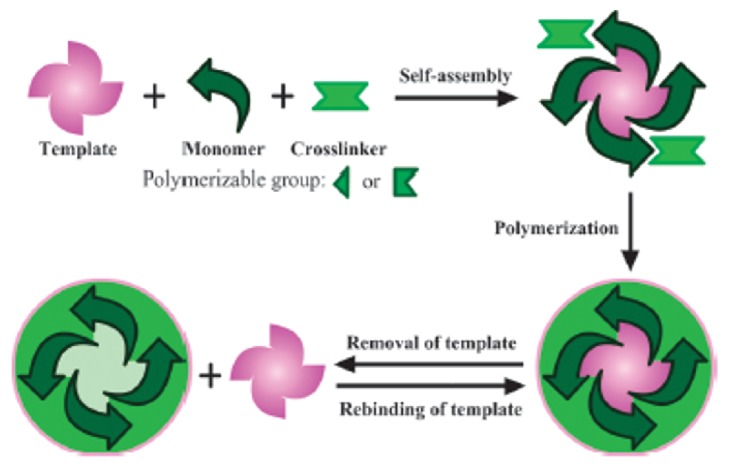
Molecular imprinting process (reproduced with permission from [25]).

Molecularly imprinted polymers (MIPs) are highly cross-linked robust materials which display excellent affinity towards target compound. For the preparation of MIPs, appropriate functional monomers and a cross-linker agent are polymerized around the target compound (template). The schematic demonstration of the molecular imprinting technique is shown in Figure 1. Due to their high affinity and selectivity for the desired compound, MIPs can be efficiently used in different application areas such as separation, catalysis, and sensor platforms [1–18]. In addition to specific molecular recognition abilities towards their target compound, MIPs can be prepared as composite nanomaterials using nanoparticles, multiwalled carbon nanotubes (MWCNTs), nanorods, quantum dots (QDs), graphene, clays in nanoscale, etc.

This paper provides the recent progress of the applications of imprinted nanocomposite materials in analytical chemistry.

## 2. MIPs in SPE Applications

Solid-phase extraction (SPE) is an efficient sample preparation technique which is one of the most widely applied approach in analytical chemistry. SPE has been first applied in 1940s [19]. Then, the progress for the current analytical applications was initiated in the 1970s. Different conventional materials such as silica based [20, 21], carbon based [22, 23], and clay based [24] resins were widely used in various applications of SPE. Although it is a popular sample preparation technique for the enrichment or extraction of the desired molecules from the complex matrices, the conventional SPE materials used in analytical applications exhibit lower selectivity towards the target molecules that lead to binding of other potentially interfering molecules existing in the sample matrices. This issue is very important especially for the complex biological samples such as urine and blood. MIP-based SPE materials that display great selectivity and binding affinity towards the target molecule/s can overcome the drawbacks of the conventional resins. In addition, MIPs preserve their stability under extreme conditions (e.g., high pressure, high temperature, and lower and higher pH).

**Figure 2:**
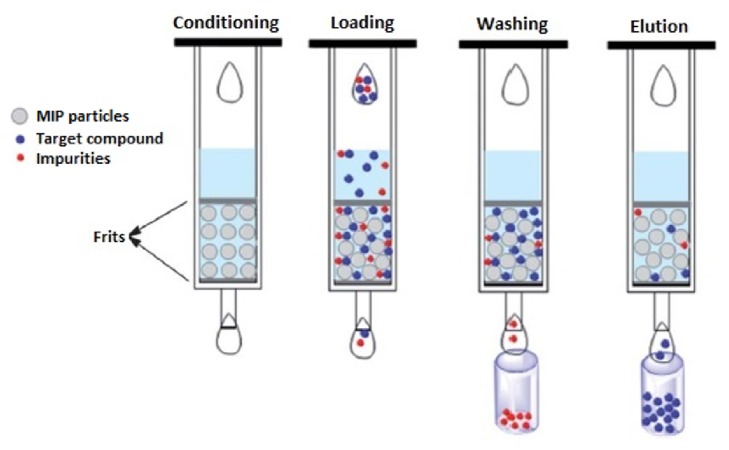
Schematic representation of SPE process (reproduced with permission from [26]).

MIP-based SPE process composed of 4 steps is schematically demonstrated in Figure 2.

Sellergren published the first SPE application of MIPs [29]. In the reported study, robust MIPs were developed for the selective extraction of drug compound pentamidine. After this successful application, many MIP-based SPE applications of various compounds in different areas were conducted and reported in the literature [30–41].

In a reported study, Su et al. developed magnetic MIP nanoparticles for the separation of bovine hemoglobin (Bhb) [114]. In their study, firstly, the preparation of magnetic Fe_3_O_4_@SiO_2_-acrylic acid (AA) nanoparticles were performed. In the second step, the preparation of BHb imprinted magnetic nanoparticles was carried out by using methacrylic acid (MAA), itaconic acid (IA), and N',N-methylenebisacrylamide as functional monomers and cross-linker, respectively. The BHb imprinted magnetic nanoparticles were efficiently used for the extraction of BHb with high binding capacity (169.29 mgg^−1^).

Viveiros et al. developed a green strategy for the preparation of selective MIPs for acetamide which is a potentially genotoxic impurity in active pharmaceutical ingredients (API) [115]. In their study, silica beads were first functionalized with 3-(Trimethoxysilyl)propyl methacrylate and then MIP layer was synthesized on the modified-silica beads using supercritical CO_2_ as the green solvent. The prepared acetamide imprinted polymers were successfully used for the extraction of acetamide from beclomethasone dipropionate which is the model API. The results showed that 100% of acetamide was removed by using selective MIPs with only very little loss of API (0.37%).

In another important study, Zhang and colleagues developed magnetic MIP-based-MWCNTs composite materials for the removal of Bisphenol A (BPA) from water matrices [116]. For this purpose, MAA was chosen as the functional monomer. The results from rebinding experiments for BPA in batch mode confirmed that the magnetic MIP-based MWCNTs have excellent affinity towards BPA and the obtained maximum binding capacity was 49.26 *μ*molg^−1^.

Yan and colleagues demonstrated the application of MIP/silica nanocomposites for the recognition of nitrocellulose [117]. The surface of the SiO_2_ particles was firstly conjugated with –OH groups and 3-(Trimethoxysilyl)propyl methacrylate (MPS) was used for the functionalization of the surface with an acrylyl groups. Then, nitrocellulose (NC) imprinted shell was synthesized on the modified-SiO_2_ particles using the functional monomer MAA and cross-linkee ethylene glycol dimethacrylate (EGDMA). The results indicated that MIP/silica nanocomposites exhibited high recognition ability towards NC with a maximum capacity of 1.7 mgmg^−1^.

**Figure 3:**
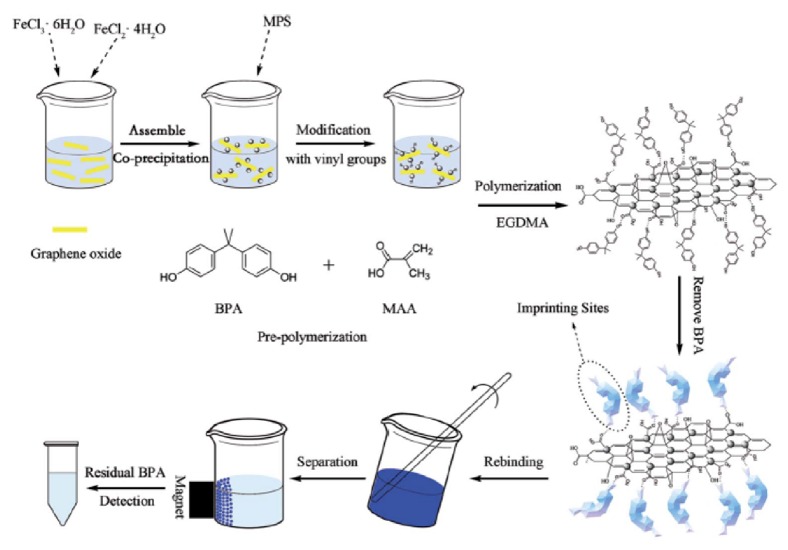
Schematic demonstration of the preparation of MIP-based magnetic graphene oxide composite towards BPA and extraction process (reproduced with permission from [27]).

In another interesting study reported by Wang and coworkers, selective extraction of BPA was successfully performed by using MIP-based magnetic graphene oxide composites [27]. For this purpose, they firstly prepared magnetic graphene oxide by using coprecipitation approach. Then, MAA (functional monomer) and BPA (template, target compound) were used for the preparation BPA imprinted magnetic graphene oxide composite. The schematic demonstration of the preparation of MIP-based magnetic graphene oxide composite towards BPA and extraction process is shown in Figure 3. The results confirmed that the prepared MIP-based magnetic graphene oxide composite displayed high selectivity towards BPA in the presence of other competing compounds such as phenol and 2,4-dichlorophenol.

Shea and his colleagues prepared imprinted hollow beads for the extraction of *β*-estradiol from tap water [118]. For this purpose, SiO_2_ nanoparticles were used as the sacrificial support. After surface modification with 3-(Trimethoxysilyl)propyl methacrylate, selective MIP shell towards *β*-estradiol was synthesized on the surface of the SiO_2_ nanoparticles using the functional monomer MAA and cross-linker EGDMA. The highest binding of *β*-estradiol was obtained within a very short time (15 min) with a maximum binding capacity of 44.5 *μ*molg^−1^.

**Figure 4:**
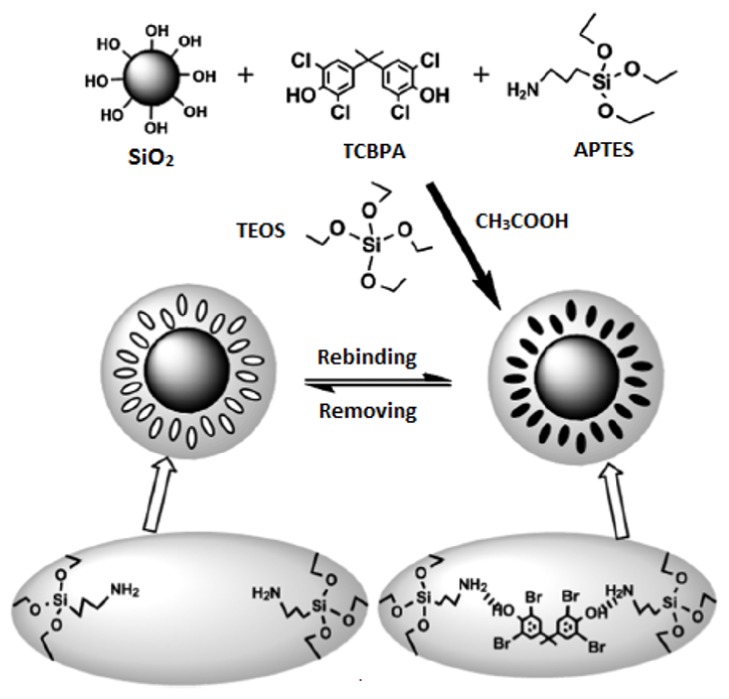
Preparation of MIP/SiO_2_ composite for TBBPA (reproduced with permission from [28]).

In another important study reported by Shen and colleagues [28], SiO_2_ particles having MIP shell were developed for the SPE of tetrabromobisphenol A (TBBPA) from river water. For this purpose, tetrachlorobisphenol A (TCBPA) was chosen as the dummy template for the preparation of MIP towards TBBPA (Figure 4). The prepared imprinted SiO_2_ particles showed fast binding kinetics (20 min) and high binding capacity (230 *μ*molg^−1^) towards the target compound TBBPA.

Guo et al. reported that magnetic graphene-based MIP composite was prepared for selective recognition of bovine hemoglobin (BHb) [119]. For this purpose, magnetic graphene was prepared in the first step. Then, MIP layer selective to BHb was prepared using the functional monomer acrylamide (AAm) which has high affinity towards BHb and cross-linker methylene bisacrylamide (MBA). Maximum binding capacity of the magnetic graphene-based MIP composite for BHb was found to be as 186.73 mgg^−1^.

**Figure 5:**
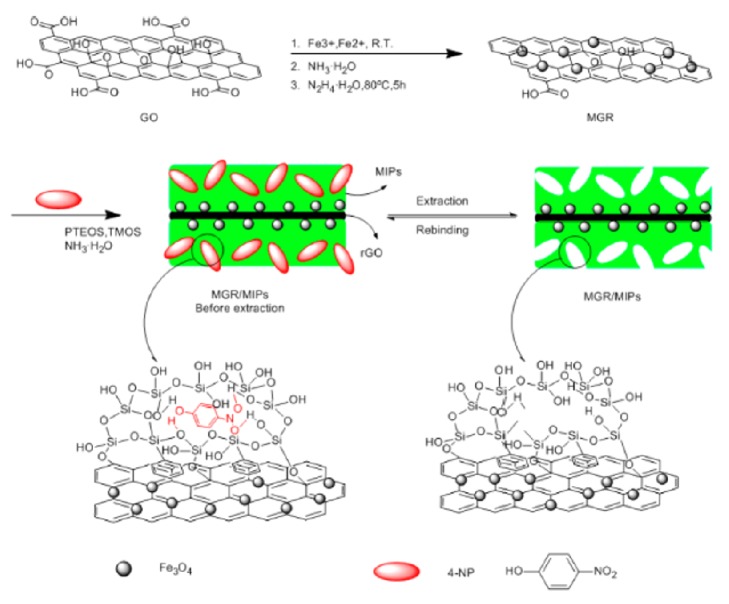
Magnetic graphene-based MIP composite towards 4-NP (reproduced with permission from [120]).

Luo et al. developed magnetic graphene-based MIP composite for the removal of 4-nitrophenol (4-NP) from aqueous solutions [120]. Fe_3_O_4_ nanoparticles were immobilized on surface of graphene sheet and magnetic graphene (MGR) was prepared in the first step. Then, MGR/MIPs composite was prepared by polymerization of phenyltriethoxysilane and tetramethoxysilane in the presence of 4-NP. The preparation of the MGR/MIPs composite is demonstrated in Figure 5. The results indicated that the prepared MGR/MIP composite displayed a great binding behavior for 4-NP with an excellent binding capacity (142 mgg^−1^).

In another research by Yang et al., core-shell magnetic MIPs were prepared for selective removal of indole from fuel oil [121]. In their research, magnetic Fe_3_O_4_ nanoparticles were synthesized by using coprecipitation technique. Then, surface of the prepared nanoparticles was coated with SiO_2_ using 3-(Trimethoxysilyl)propyl methacrylate. In the final step, the functional monomer MAA and EGDMA (cross-linker) were polymerized on the surface of the modified magnetic nanoparticles for the preparation of selective MIP shell towards indole. The results confirmed that the prepared magnetic MIP composite displayed excellent recognition ability towards the target compound indole. The binding capacity of the composite for indole was obtained as 50.25 mgg^−1^.

In another interesting study [122], Cao et al. prepared MIP-based-MWCNTs for the SPE of perfluorooctanoic acid from aqueous matrices. In their study, they used the functional monomer AAm for the preparation of MIP. After characterization studies, the prepared MIP-based-MWCNTs as composite SPE materials were successfully used for the selective removal of perfluorooctanoic acid from aqueous matrices. The obtained results confirmed that the binding equilibrium was obtained in 80 min. The determined binding capacity was 12.4 mgg^−1^.

Table 1 shows the recent examples of the SPE applications of nanostructured MIP-based composites.

## 3. MIPs in Sensor Applications

MIP-based sensors can be categorized into 3 basic groups: electrochemical, spectroscopic, and piezoelectric sensors. In the following sections, recent examples of MIP-based sensors are briefly explained.

### 3.1. MIP-Based Electrochemical Sensors

In electrochemical detection, the reaction generally leads to a change of current (amperometric), potential (potentiometric), or conductivity (conductometric) [129]. Selectivity and sensitivity are crucial parameter for electrochemical sensors. Surface modification of electrodes in electrochemical sensors by immobilization of recognition components is an efficient approach to obtain a high binding of target compound with good selectivity and good response. The surface modification of electrodes in the design and preparation of electrochemical sensors has firstly been reported by Itaya and Bard in 1978 [130]. Since then, many studies on the design and development of electrochemical sensors in different application areas have been reported.

In a reported study [131], an electrochemical sensor having MIP film for the theophylline recognition was prepared by Kan and colleagues. In their study, the functional monomer o-phenyldiamine was used as the functional monomer for the preparation of MIP film. After MIP film preparation on the glassy carbon electrode surface, gold nanoparticles were immobilized onto MIP film. The prepared MIP-based electrochemical sensor was characterized by SEM and binding behavior towards theophylline was tested using CV, differential pulse voltammetry, and EIS. The detection limit for theophylline was found to be as 1.0×10^−7^molL^−1^.

**Figure 6:**
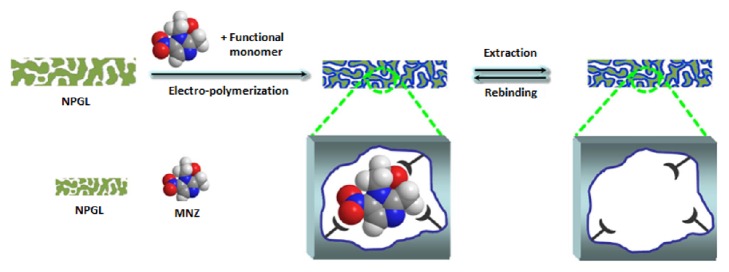
Preparation of MIP-based electrochemical sensor towards MNZ (reproduced from Li et al. (2015) [under the Creative Commons Attribution License/public domain]).

Li and colleagues developed an electrochemical sensor composed of nanoporous gold leaf (NPGL) electrode having selective MIP layer for the detection of metronidazole (MNZ) [132]. The preparation of the MIP-based electrochemical sensor towards MNZ is schematically shown in Figure 6. The experimental results confirmed that the developed electrochemical sensor has excellent binding affinity towards MNZ in fish tissue samples. The detection limit was obtained as 1.8×10^−11^molL^−1^.

In a study reported by Gupta and Goyal, a new graphene/MIP composite sensor for the determination of melatonin in biological samples was prepared [133]. For this purpose, MIP layer was prepared on the glassy carbon electrode (GCE) surface by copolymerization of 4-amino-3-hydroxy-1-naphthalenesulfonic acid and melamine around the template melatonin. The optimization studies for MIP layer formation were carried out changing the parameters such as monomer/template ratio and time. After characterization of the prepared composite electrochemical sensor for melatonin by SEM and EIS, the binding performance of the sensor towards target melatonin was carried out by using square wave voltammetry and cyclic voltammetry. The obtained results showed that efficient recognition of melatonin in plasma samples was successfully achieved. The determined detection limit was 0.006 *μ*M.

**Figure 7:**
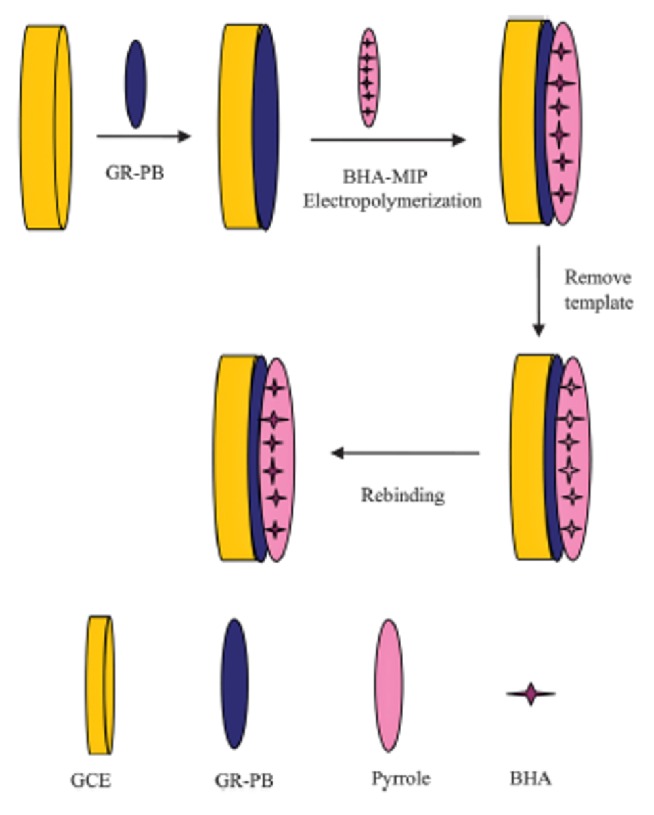
GR-PB/MIP-based composite electrochemical sensor towards BHA (reproduced with permission from [123]).

Cui et al. prepared graphene-Prussian blue (GR-PB)/MIP-based composite electrochemical sensor for selective detection of butylated hydroxyanisole (BHA) in food samples [123]. In this study, MIP film was synthesized on the surface of GCE having GR-PB by electropolymerization of the functional monomer pyrrole and the template BHA (Figure 7). The prepared composite sensor was characterized by SEM, cyclic voltammetry (CV), electrochemical impedance spectroscopy (EIS), and chronoamperometry. The results obtained from the experiments for the sensor performance showed that immobilization of GR and PB onto the GCE increased the sensor sensitivity and the response towards target BHA. The prepared composite electrochemical sensor showed a linear response towards BHA (9 x 10^−8^ M to 7 x 10^−5^ M) and the detection limit was calculated as 7.63 x 10^−8^ M.

In an interesting study published by Prasad and colleagues, a composite electrochemical sensor composed of MIP film and MWCNTs was prepared for the detection of L-histidine [134]. MIP film selective to L-histidine was prepared by polymerization of 2-acryl amidoethyl dihydrogen phosphate (functional monomer) and EGDMA (cross-linker). In the first step, the functional monomer was interacted with Cu (II). Then, polymerization was performed in the presence of Cu (II)-functional monomer-template complex. The prepared MIP-based electrochemical sensor showed enantioselectivity towards L-histidine and the detection limit was found to be as 1.980 ngmL^−1^. However, cross-reactivity studies of the prepared sensor for potentially interfering compounds in the sample such as L-phenylalanine, D- histidine, L- and D-tryptophan, L-tyrosine, L-methionine, L-alanine, L-glycine, L-proline, urea, dopamine, creatinine, uric acid, L-glutamic acid, and L-ascorbic acid were also performed. The results confirmed that the prepared composite electrochemical sensor exhibited very low response towards these interfering compounds.

A carbon nanotube (CNT)/Graphene (GP)/MIP-based composite electrochemical sensor for the detection of bovine serum albumin (BSA) was developed by Chen and colleagues [15]. For this purpose, carbon electrode (CE) was modified with GP in the first step. Then, CNT was prepared on the surface of modified CE with GP. In the final step, MIP membrane was synthesized on the CNT/GP/CE by electrodeposition of aniline in the presence of template BSA. The prepared sensor was successfully applied for sensitive recognition of BSA in human serum with a detection limit of 6.2 x 10^−11^gmL^−1^.

Wang and coworkers reported the preparation of CdS quantum dot/graphene/MIP-based electrochemical sensor for selective recognition of 4-aminophenol in water samples [135]. In their study, fluorine-doped tin oxide (FTO) electrode was modified with CdS quantum dots and graphene (GR). Then, a MIP film selective to target compound 4-aminophenol was prepared by electropolymerization. The results confirmed that the developed electrochemical sensor specifically binds the target 4-aminophenol. The response of the sensor towards 4-aminophenol was linear in the concentration range of 5.0 x 10^−8^ M to 3.5 x 10^−6^ M and the determined detection limit was 2.3 x 10^−8^ M.

### 3.2. MIP-Based Spectroscopic Sensors

MIP-based spectroscopic sensors can be divided into 3 categories. These are MIP-based-fluorescence sensors, MIP-based-chemiluminescence sensors, and MIP-based-SPR sensors. In the fluorescence based molecular recognition of the target compound, fluorescence functional monomers are chosen for the fabrication of sensor platforms based on molecular imprinting technique [136]. When the target compound binds to the sensor, fluorescence intensity increases or decreases depending on the sensor design.

In a significant research reported by Zhang and colleagues [137], CdSe/ZnS quantum dots (QDs) coated with MIP film which shows fluorescence feature were synthesized for the sensitive recognition of carbaryl in cabbage and rice samples. For this purpose, MAA was used as the functional monomer for the synthesis of MIP layer on the QDs surface modified with the ionic liquid. The obtained results from the fluorescence measurements showed that the fluorescence sensor composed of QD_S_-MIP exhibited high recognition ability towards carbaryl in the presence of metolcarb and isorcarb which are analogues of carbaryl.

Mehrzad-Samarin et al. developed a novel graphene QDs embedded silica MIP-based fluorescence sensor for the selective recognition of metronidazole [138]. The prepared sensor showed a linear response towards metronidazole in the range between 0.2 *μ*M and 15*μ*M. The determined detection limit was 0.15 *μ*M.

Li and coworkers developed magnetic silica nanoparticles having selective MIP shell for the recognition of Rhodamine B from aqueous samples [139]. In this study, magnetic silica nanoparticles were coated with MIP layer using nitrobenzoxadiazole which is a fluorophore molecule. The obtained results confirmed that the efficient detection of Rhodamine B in aqueous samples was performed by using MIP-based magnetic silica nanoparticles. The maximum binding of Rhodamine B was obtained in 60 min with a high binding capacity (29.64 mgg^−1^).

In another study reported by Jalili and Amjadi [140], MIP/green emitting carbon dot composite was prepared for the selective recognition of 3-nitrotyrosine which is a biomarker for various diseases such as rheumatoid arthritis, Alzheimer, atherosclerosis, osteoarthritis, and cardiovascular diseases. The prepared MIP-based composite fluorescence sensor was efficiently used for the selective recognition of 3-nitrotyrosine in human serum samples in the concentration range from 0.05 to 1.85 *μ*M and the detection limit was obtained as 17 nM.

**Figure 8:**
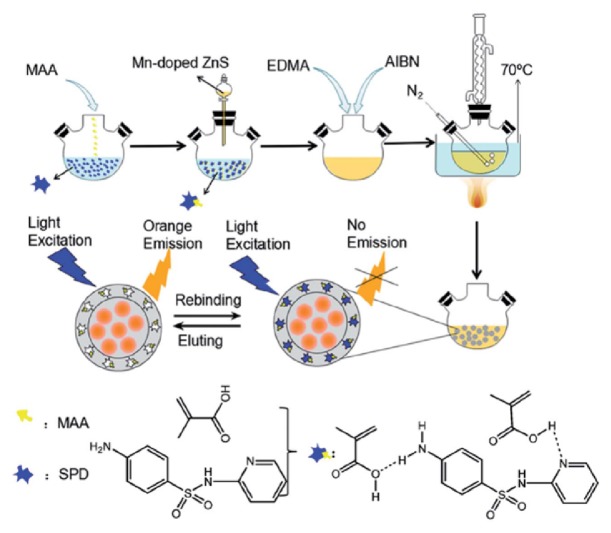
The preparation of ZnS QDs/MIP-based fluorescence nanosensor towards sulfapyridine (reproduced with permission from [124]).

The research group of Hu was developed a ZnS QDs/MIP-based fluorescence nanosensor for the sensitive detection of sulfapyridine in tap water samples [124]. For this purpose, Mn-doped ZnS QDs was used as the fluorescence core and MIP shell was prepared on the surface of the QDs by using the functional monomer MAA, cross-linker EDMA, initiator AIBN, and template sulfapyridine (SPD). The schematic demonstration of the preparation of ZnS QDs/MIP-based fluorescence nanosensor towards sulfapyridine is shown in Figure 8. The prepared ZnS QDs/MIP-based fluorescence nanosensor exhibited high recognition ability towards SPD with a detection limit of 0.5 *μ*M.

Chemiluminescence is another efficient approach that is used for the investigation of the recognition performance of MIP-based spectroscopic sensor systems. In this approach, a chemiluminescence system is chosen and selective MIPs are integrated to this system. When target compound binds to the MIP-based sensor, chemiluminescence emission is generated. The amount of the emission depends on the amount of bound target compound to the sensor surface.

**Figure 9:**
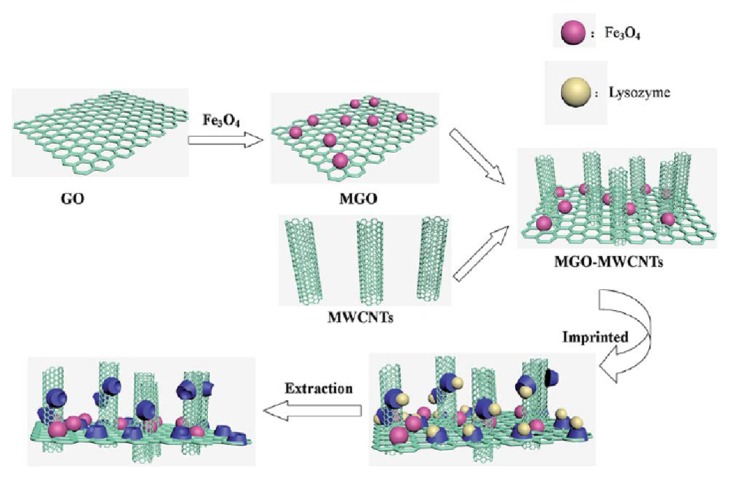
Schematic depiction of the preparation of magnetic GO/MWCNTs/MIP-based chemiluminescence nanosensor towards lysozyme (reproduced with permission from [125]).

In a study conducted by Wang and coworkers [125], a magnetic graphene oxide (GO)/MWCNTs/MIP-based chemiluminescence nanosensor was developed for the sensitive detection of lysozyme in egg samples.  Figure 9 shows the schematic demonstration of the construction of the magnetic GO/MWCNTs/MIP-based chemiluminescence nanosensor towards lysozyme. The developed chemiluminescence nanosensor displayed high sensitivity towards lysozyme. The obtained detection limit was 1.9 x 10^−9^  gmL^−1^.

SPR-based sensor platforms are also popular recognition systems. SPR technique relies on the measurement of the changes in refractive index of thin layer on the metal surface. The recognition element on the surface of the sensor is usually gold or silver coated with thin film. Therefore, uniform film layer is synthesized on the surface of MIP-based-SPR sensors.

Many studies were published on the development of MIP-based SPR sensors and their applications. For example, the group of Piletsky developed a molecularly imprinted nanoparticle-based SPR sensor system for the sensitive detection of diclofenac in aqueous solutions [141]. For this purpose, diclofenac imprinted nanoparticles were synthesized by using styrene as the functional monomer, EGDMA and trimethylolpropane trimethacrylate (TRIM) as cross-linkers, and pentaerythritol tetrakis (3-mercaptopropionate) as the chain transfer agent. Then, the surface of the SPR sensor was activated by using N-Hydroxysuccinimide (NHS) and 1-Ethyl-3-(3-dimethylaminopropyl)-carbodiimide (EDC). After activation step, the prepared diclofenac imprinted nanoparticles were immobilized onto the surface of the sensor. The sensitive detection of diclofenac was successfully achieved in the concentration range from 1.24 to 80 ngmL^−1^. The selectivity of the SPR sensor towards diclofenac in the presence of propranolol and vancomycin was also studied. The experimental data confirmed that the sensor exhibited high selectivity towards diclofenac.

In another interesting study [142], Ashley et al. prepared a MIP-based SPR nanosensor for the sensitive detection of *α*-casein cleaning in place (CIP) wastewater samples. For this purpose, immobilization of the target protein *α*-casein (template) on the surface of glass nanobeads was carried out in the first step. Then, MIP nanoparticles were prepared by using N-(3-aminopropyl)-methacrylamide, the functional monomer acrylic acid, and cross-linker N,N′-methylenebis(acrylamide). Finally, *α*-casein imprinted nanoparticles were incorporated onto the SPR sensor surface. The results confirmed that the developed MIP-based SPR nanosensor showed excellent selectivity and affinity (K_D_ ~ 10 x 10^−9^ M) towards target protein *α*-casein. The detection limit was obtained as 127 ngmL^−1^.

### 3.3. MIP-Based Piezoelectric Sensors

Quartz crystal microbalance (QCM) is another popular an analytical technique that displays high sensitivity to mass changes on the sensor surface. Many examples on different applications of QCM sensor systems have been reported in the literature and some examples are briefly described in the following.

Eren et al. [143] developed a QCM sensor system having MIP layer for the detection of lovastatin in red yeast rice. MIP layer was prepared on the surface of allyl mercaptan modified-gold electrode by the polymerization of HEMA, MAAsp as the functional monomers, and cross-linker EGDMA in the presence of template compound lovastatin. The developed QCM sensor having MIP layer was successfully applied for the sensitive recognition of lovastatin in red yeast rice samples. The limit of detection of the prepared QCM sensor towards lovastatin was found to be as 0.030 nM.

A QCM having MIP layer towards profenofos was developed by Gao and coworkers [144]. For this purpose, they used MAA as the functional monomer for the synthesis of profenofos imprinted MIP layer on the surface of gold electrode modified with 11-mercaptoundecanoic acid. The developed QCM sensor with MIP layer showed high sensitivity towards the target compound profenofos in aqueous solutions with an excellent detection limit of 2.0 x 10^−7^mgmL^−1^.

**Figure 10:**
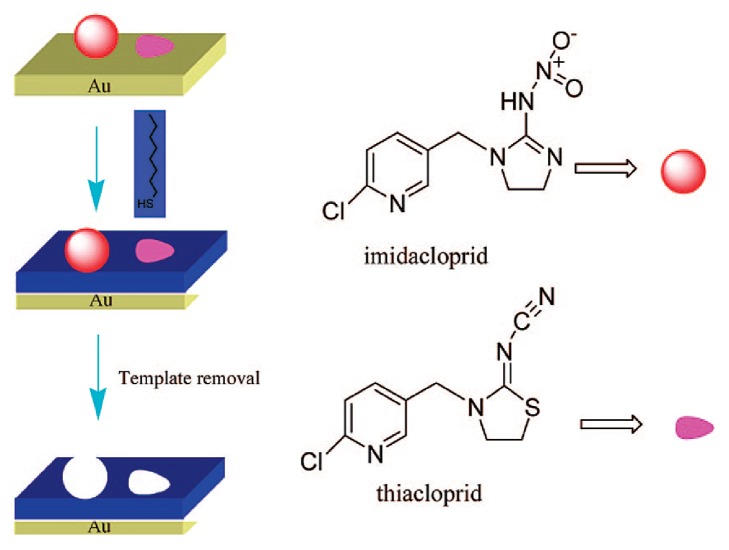
MIP-based QCM sensor towards imidacloprid and thiacloprid (reproduced with permission from [126]).

In another study [126], Bi and Yang prepared a QCM sensor platform bearing MIP layer for the detection of pesticide compounds imidacloprid and thiacloprid in celery juice. For this purpose, the immobilization of the target compounds on the surface of the gold chip was performed in the first step. Then, self-assembly of alkanethiols around the target compounds was carried out and the template removal was performed by using EtOH. The demonstration of the QCM sensor bearing MIP layer towards imidacloprid and thiacloprid is shown in Figure 10. The developed sensor system displayed good recognition behavior towards the target compounds imidacloprid and thiacloprid. It has also been noted that these sensor systems are promising and have the potential to detect pesticide residues in aqueous solutions and vegetables.

In another interesting study [145], the detection of metolcarb in food and beverage samples such as cabbage, pear, and apple juice was carried out by using MIP-based QCM sensor. The results indicated that the developed QCM sensor displayed a linear response towards metolcarb in the range between 5 and 70 *μ*gL^−1^. The detection limit was obtained as 2.309 *μ*gL^−1^.

Table 2 shows the recent examples of nanostructured MIP-based composites in sensor applications.

## 4. MIPs in Catalytic Applications

Enzyme-like catalysts are also popular application of imprinted nanomaterials in biomimetic catalysis. For the preparation of enzyme-like catalysts based on molecular imprinting approach, appropriate functional monomers are chosen and incorporated in the polymeric network by choosing the substrate of enzyme (as the template compound) or the transition state analogue (TSA) of the target reaction. After removal of the template from the polymeric network, the obtained imprinted nanomaterial behaves as enzyme-like catalyst towards the desired chemical or biochemical reaction. Some examples reported in the literature are briefly discussed in the following.

**Figure 11:**
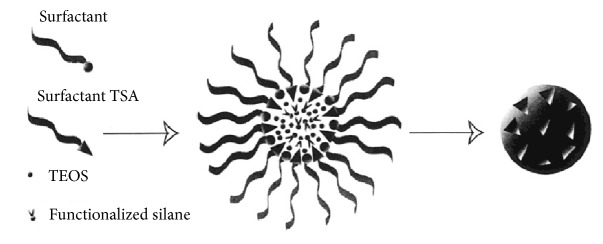
Preparation on MIP-based silica nanoparticles (reproduced with permission from [127]).

Markowitz and coworkers developed MIP-based silica nanocomposites for the selective hydrolysis of substrates of chymotrypsin and trypsin [127]. For this purpose, a TSA of *α*-chymotrypsin was used as the template compound for the preparation of *α*-chymotrypsin-like nanocatalyst (Figure 11). The silane groups conjugated with the amino acids which exist in the catalytic center of the *α*-chymotrypsin were used for the preparation of silica nanoparticles. The activity of the prepared MIP-based silica nanocomposites was performed by monitoring the hydrolysis of the substrates succinyl-Ala-Ala-Pro-Phe-*p*-nitroanilide and benzoyl-DL-arginine-*p*-nitroanilide. The developed imprinted nanocatalyst showed great enantioselective hydrolytic activity towards the substrate compounds.

**Figure 12:**
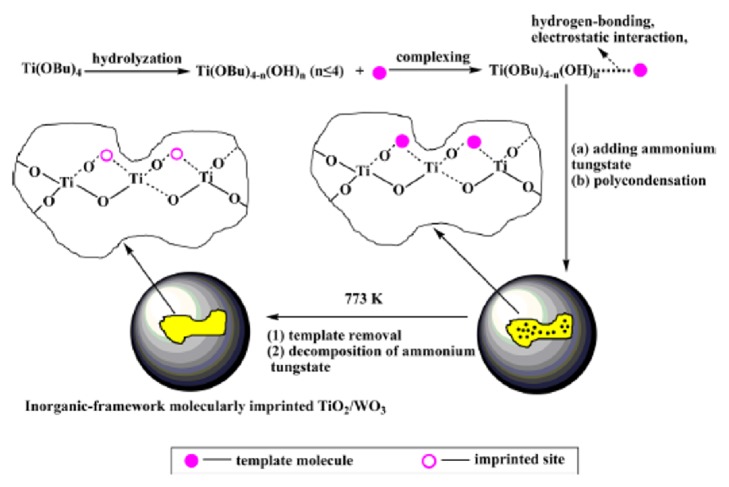
TiO_2_/WO_3_/MIP-based composite nanocatalyst towards 2-nitrophenol and 4-nitrophenol (reproduced with permission from [128]).

Luo et al. developed a TiO_2_/WO_3_/MIP-based composite nanocatalyst for the efficient degradation of 2-nitrophenol and 4-nitrophenol [128]. One-step sol-gel method was applied for the preparation of composite nanocatalyst by using tetrabutyl orthotitanate which was chosen as the functional monomer precursor and titanium source. The schematic representation of the prepared composite nanocatalyst is shown Figure 12.

The obtained results indicated that the photocatalytic activity of the prepared TiO_2_/WO_3_/MIP-based composite nanocatalyst towards the target compounds is 2 times higher than its corresponding nonimprinted catalyst.

In a study reported by Bonomi et al. [146], catalytic imprinted nanogels were synthesized for the Kemp elimination reactions. The functional monomer 4-VP and template compound 5-nitro indole were used for the synthesis of imprinted nanogels. The results showed that the prepared 5-nitro indole imprinted nanogels exhibited high catalytic activity towards the substrate 1,2-benzisoxazole. Substrate selectivity of the prepared catalytic nanogels was also investigated using 5-Cl-benzisoxazole which is a substrate analogue. The catalytic nanogels displayed lower affinity towards 5-Cl-benzisoxazole compared to the substrate 1,2-benzisoxazole.

In another interesting study, Zhou and colleagues prepared a molecularly imprinted TiO_2_ photocatalyst having thiol groups for the efficient removal of 2,4-dinitrophenol from wastewater [147]. MIP-based TiO_2_ photocatalyst was prepared in water as a green solvent using o-phenylenediamine as the functional monomer. The results confirmed that the prepared MIP-based green photocatalyst displayed excellent selectivity and degradation activity towards 2,4-DNP in wastewater.

## 5. Conclusions

The growing number of published researches in which nanostructured composite MIPs have been used for different applications showed that these are promising materials for the selective extraction, sensing, and catalysis. The reported studies described in this review highlight the recent progress in SPE, sensors, and catalytic systems using nanostructured composite MIPs over the past years. Composite MIPs in nanoscale as promising materials provide a new approach for the selective SPE and sensors towards target molecules in complex matrices. On the other hand, these materials offer new routes to control aspects that determine the stereo-chemical outcome of a catalysis reaction.

## Figures and Tables

**Table 1:** Recent examples of nanostructured MIP-based composites in SPE applications.

**Reference**	**Nanocomposite composition**	**Analyte**	**Sample**
**Applications to environmental samples**			

[42]	Magnetic nanoparticles coated with MIP having the functional monomer 4-vinyl pyridine (4-VP)	Cr^6+^	Water

[43]	Silica-MIP composite prepared by grafting method	[UO_2_]^2+^	Water

[44]	Magnetic nanoparticles coated with MIP having –NH groups	Co^2+^	Water

[45]	Chitosan-MIP magnetic nanocomposite	Ni^2+^	Water

[46]	Silica-MIP monolithic composite column	*α*-cypermethrin	Soil

[47]	Cu(II)-mediated silica fiber-MIP composite	Thiabendazole	Soil

[48]	Magnetic nanoparticles coated with MIP having MAA and 4-VP as functional monomers	Methyl parathion	Soil

[49]	Magnetic nanoparticles coated with MIP prepared by using the functional monomer gelatin	17*β*-estradiol	Water

**Applications to clinical samples**			

[50]	Magnetic SiO_2_ nanoparticles having MIP shell prepared by using the functional monomer MAA	Amitriptyline	Human plasma and urine

[51]	Magnetic SiO_2/_/MIP/chitosan biocomposite	Baclofen	Human urine

[52]	Magnetic nanoparticles having MIP shell prepared by using the functional monomer MAA	Rizatriptan	Human urine

[53]	Magnetic nanoparticles having MIP shell prepared by using the functional monomer MAA	Paracetamol	Human plasma

[54]	Optical fiber coated with MIP prepared by sol-gel method	Caffeine	Human serum

[55]	Magnetic nanoparticles having MIP shell prepared by using the functional monomer AAm	Protoberberine alkaloids	Rat plasma

[56]	Magnetic CNTs coated with MIP having carboxyl groups	Catecholamines	Human plasma

[57]	Magnetic nanoparticles having MIP shell prepared by using the functional monomer MAA	Tizanidine	Human urine

[58]	Magnetic nanoparticles coated with MIP having aminoimide as the functional monomer	Codeine	Human urine

[59]	Silica-MIP composite having AAm, MAA and 4-VP as functional monomers	Baicalin	Rat tissues

**Applications to food and beverage samples**			

[60]	Carbon QDs-doped MIP monolithic column bearing the functional monomer MAA	Aflatoxin B1	Peanut

[61]	Magnetic nanoparticles having MIP shell bearing the functional monomer MAAm	Dimethoate	Olive oil

[62]	Magnetic MWCNTs having MIP bearing the functional monomer MAA	Melamine	Milk

[63]	Magnetic nanoparticles having MIP shell prepared by using ethyl paraoxon as the dummy template	organophosphorus pesticide	Red wine

[64]	Magnetic nanoparticles coated with MIP having AA as the functional monomer	Imidacloprid	Honey and eggplant

[65]	Magnetic nanoparticles coated with MIP having MAAm and N-3,5-bis(trifluoromethyl) phenyl-N'-4-vinylphenyl urea as functional monomers	Citrinin	Rice

[66]	Magnetic nanoparticles having MIP shell prepared by using the functional monomer MAA	Malachite green	Fish

[67]	Magnetic nanoparticles coated with MIP having oleic acid	Oxytetracycline	Honey, Egg

[68]	Carbon dots coated with MIP prepared by sol–gel method	Sterigmatocystin	Grain

[69]	Magnetic nanoparticles coated with MIP having dopamine as the functional monomer	Gallic acid	Grape, Apple, Peach and Orange juices

[70]	Magnetic nanoparticles coated with MIP having vinyl groups	Ni(II)	Cucumber, Cantaloupe, Apple, Nectarine, Green beans, Fenugreek, Dill, Tuna fish

[71]	Silica nanoparticles having MIP shell bearing the functional monomer MAA	Ofloxacin	Milk

[72]	Magnetic nanoparticles having MIP shell bearing the functional monomer dopamine	Diethylstilbestrol	Milk

[73]	Magnetic nanoparticles having MIP shell bearing the functional monomer AAm	*β*-agonists	Pork

[74]	Magnetic nanoparticles having MIP shell bearing the functional monomer MAA	Chloramphenicol	Honey

**Table 2:** Recent examples of nanostructured MIP-based composites in sensor applications.

**Reference**	**Nanocomposite composition**	**Analyte**	**Sample**
**Electrochemical sensors**			

[75]	Pencil graphite electrode coated with molecularly imprinted polypyrrole	Methylimidazole	Serum

[76]	Glassy carbon electrode modified with graphene/ Au nanoparticles/MIP composite	Colchicine	Serum and pharmaceuticals

[77]	Glassy carbon electrode modified with graphene/Ag nanoparticles/MIP composite	Creatinine	Saliva and serum

[78]	Glassy carbon electrode coated with CNT/MIP composite	Tramadol	Urine

[79]	Carbon paste electrode coated with MIP	Zn^2+^	River water, urine and blood

[80]	Graphite electrode coated with MIP	Azithromycin	Drug

[16]	Glassy carbon electrode coated with graphene/ CNT/MIP composite	Propyl gallate	Vegetable oil

[81]	Carbon paste electrode coated with CNT/MIP nanoparticle composite	Meloxicam	Plasma

[82]	Glassy carbon electrode coated with MIP/Pd nanoparticles composite	Norepinephrine	Urine

[83]	Carbon paste electrode coated with MIP	Famciclovir	Drug

[84]	Glassy carbon electrode coated with graphene/MIP membrane composite	Artemisinin	Plant extract

[85]	Glassy carbon electrode coated with MIP/Au nanoparticles composite	Estradiol	Milk

[86]	Interdigitated electrode coated with CNT/MIP composite	Cotinine	Organic solutions

[87]	Glassy carbon electrode coated with CNT/MIP/Pt nanoparticles composite	Tartrazine	Beverages

[88]	Carbon electrode coated with graphene/MIP/Ni nanoparticles composite	Tetrabromo bisphenol A	Tap water, rain and lake water

[89]	Carbon electrode coated with graphene/MIP/Ag nanoparticles composite	Bisphenol A	Plastic samples and soil samples

[90]	Carbon paste electrode coated with MIP	Trinitrotoluene	Tap water and sea water

**Spectroscopic sensors**			

[91]	CdTe QDs embedded-SiO_2_ particles coated with MIP layer	Neomycin	Pork, swine liver, swine kidney, fish meat, fish liver, chicken meat, chicken kidney and milk

[92]	CdSe/ZnS QDs having MIP shell	Trichlorfon	Spinach and rape samples

[93]	Luminescent magnetic MIP nanoparticles having LaVO_4_:Eu^3+^ nanocrystals	Diazinon	Aqueous solutions

[94]	Chemiluminescent Fe_3_O_4_@SiO_2_ magnetic nanoparticles coated with MIP layer	Sulfadiazine	Urine

[95]	SPR sensor having MIP layer bearing the functional monomer MAA	Clenbuterol	Aqueous solutions

[96]	SPR sensor having MIP layer bearing the functional monomer MAA	Ametryn	Soybean and rice

[97]	ZnS QDs doped with Mn/MIP composite	Domoic acid	Shellfish

[98]	ZnO nanorods coated with molecularly imprinted poly(ethylene-co-vinylalcohol)	Melatonin	Urine

[99]	Magnetic nanoparticles having MIP layer bearing the functional monomer MAA	Mefenamic acid	Aqueous solutions

[100]	SPR sensor surface having MIP layer bearing the functional monomer MAA	L-nicotine	Aqueous solutions

[101]	Graphene QDs coated with MIP layer	Dopamine	Serum and Urine

**Piezoelectric sensors**			

[102]	QCM sensor surface coated with 1,3,5-pentanetricarboxylic acid imprinted film	Domoic acid	Mussel extracts

[103]	QCM sensor surface coated with MIP film having styrene/DVB copolymer	Terpenes	Herbs

[104]	QCM sensor surface coated with MIP film having 1,3,5 trisacrylamide 2,4,6 triazine as the functional monomer	Folic acid	Aqueous solutions

[105]	QCM sensor having MIP layer bearing the functional monomer MAA	Ni^2+^ and Cu^2+^	Aqueous solutions

[106]	QCM sensor surface coated with polythiophene MIP film	Pinacolyl methyl phosphonate	Aqueous solutions

[107]	QCM sensor surface having MIP/Au nanoparticles/ poly(o-aminothiophenol) membrane	Ractopamine	Swine feed

[108]	QCM sensor having MIP layer bearing the functional monomer AA	Glucose	Aqueous solutions

[109]	QCM sensor having MIP layer bearing the functional monomer MAA	Microcystin	Lake water

[110]	QCM sensor having MIP layer bearing the functional monomer 1-Vinyl-2-pyrrolidone	Heparin	Plasma

[111]	QCM sensor having MIP layer bearing the functional monomer MAA	Methimazole	Urine

[112]	QCM sensor having MIP layer bearing zinc acrylate as the functional monomer	Human serum albumin	Human serum

[113]	QCM sensor having MIP layer bearing 3-aminopropyltriethoxysilane as the functional monomer	Enrofloxacin	Milk, egg, chicken muscle and pork
